# Challenges in quantifying genome erosion for conservation

**DOI:** 10.3389/fgene.2022.960958

**Published:** 2022-09-26

**Authors:** Mirte Bosse, Sam van Loon

**Affiliations:** ^1^ Amsterdam Institute for Life and Environment (A-LIFE), Section Ecology and Evolution, Vrije Universiteit Amsterdam, Amsterdam, Netherlands; ^2^ Animal Breeding and Genomics, Wageningen University and Research, Wageningen, Netherlands

**Keywords:** conservation genomics, runs of homozygosity, genetic load, inbreeding, conservation, genetic diversity

## Abstract

Massive defaunation and high extinction rates have become characteristic of the Anthropocene. Genetic effects of population decline can lead populations into an extinction vortex, where declining populations show lower genetic fitness, in turn leading to lower populations still. The lower genetic fitness in a declining population due to a shrinking gene pool is known as genetic erosion. Three different types of genetic erosion are highlighted in this review: overall homozygosity, genetic load and runs of homozygosity (ROH), which are indicative of inbreeding. The ability to quantify genetic erosion could be a very helpful tool for conservationists, as it can provide them with an objective, quantifiable measure to use in the assessment of species at risk of extinction. The link between conservation status and genetic erosion should become more apparent. Currently, no clear correlation can be observed between the current conservation status and genetic erosion. However, the high quantities of genetic erosion in wild populations, especially in those species dealing with habitat fragmentation and habitat decline, may be early signs of deteriorating populations. Whole genome sequencing data is the way forward to quantify genetic erosion. Extra screening steps for genetic load and hybridization can be included, since they could potentially have great impact on population fitness. This way, the information yielded from genetic sequence data can provide conservationists with an objective genetic method in the assessment of species at risk of extinction. However, the great complexity of genome erosion quantification asks for consensus and bridging science and its applications, which remains challenging.

## 1 Alarming decline in biodiversity

The Anthropocene is seeing a tremendous amount of defaunation. In the last 500 years alone, at least 322 species of terrestrial vertebrates have gone extinct, with remaining population sizes showing an average decline of 25%. Invertebrates show a similar pattern, with two thirds of monitored populations showing a mean 45% abundance decline (Dirzo et al., 2014). Recent research even suggests we are currently in the middle of the sixth mass extinction (Barnosky et al., 2011; Ceballos et al., 2017). Despite ongoing activities to halt biodiversity loss, we are still losing many species at an increasing rate (Turvey & Crees, 2019; Gumbs et al., 2020). Population decline has many causes, such as habitat fragmentation, introduction of non-native species, climate change, pollution and exploitation (Barnosky et al., 2011), and strengths of these threats heavily depend on local context and metrics used (Bellard et al., 2022). Many threatened populations are critically small, and protecting their habitat is not sufficient to keep such populations viable (Baker, 2007).

### 1.1 The role of genetics in species extinction

**FIGURE 1:**
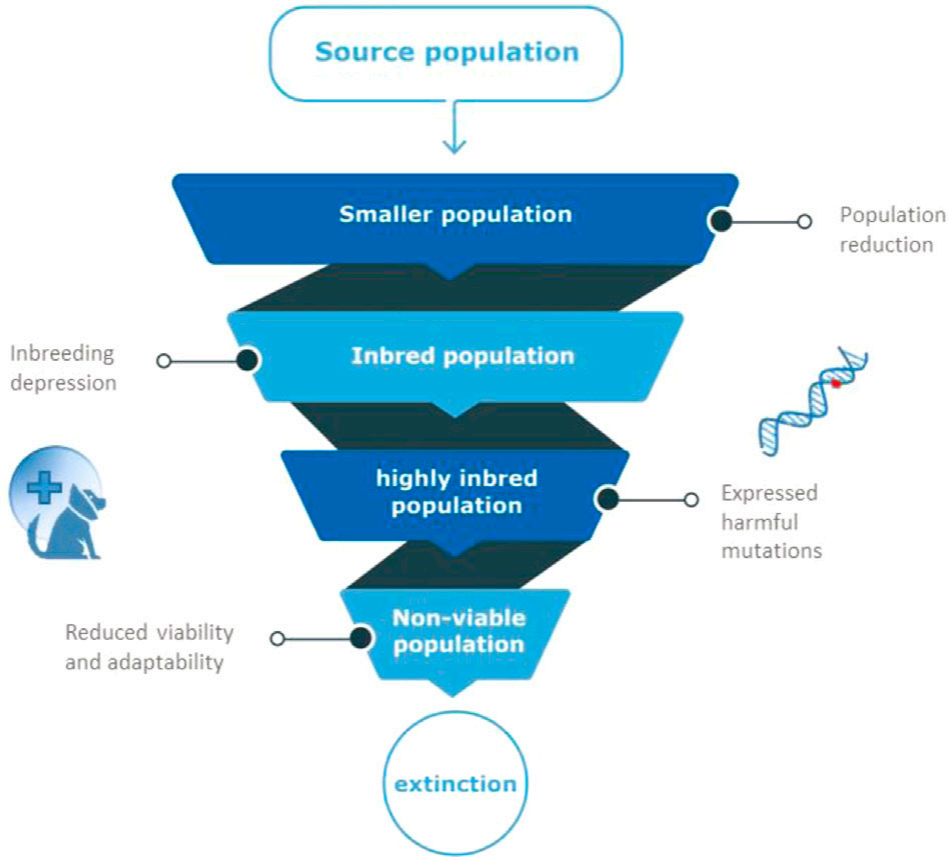
The extinction vortex. When population size is reduced, related animals produce inbred offspring. Inbred animals will have reduced fertility and survival because of harmful mutations that appear in homozygous state in inbred individuals (inbreeding depression). Due to the fixation of such harmful mutations in small populations, overall viability of the population is reduced, leading to more inbreeding, a non-viable population and eventual extinction.

The effects of population decline are numerous. Inbreeding is another threat to such marginalized populations (Lynch et al., 1995), because of increased risk of genetic defects and exposure of recessive harmful mutations (Keller & Waller, 2002). Inbreeding is the inheritance of identical copies of genetic material from related parents, and causes long homozygous regions in the genome of the offspring (Runs Of Homozygosity Curik et al., 2014). The decline in fitness observed in inbred progeny, relative to outbred progeny is known as “*inbreeding depression”* (Keller & Weller, 2002). Animals with inbreeding depression have reduced fitness that manifests as reduced survivorship or fecundity, influencing population survival under environmental change in the long-term (Willi & Hoffmann, 2009; Åkesson et al., 2016; Stoffel et al., 2021). Although the relative contribution of genetics-related causes of population collapse is still debated, the common consensus currently seems to be that it can contribute to species extinction (Spielman et al., 2004; Frankham, 2005; Brook et al., 2006; Agrawal & Whitlock, 2012; Genereux et al., 2020). The spiral of a decreasing effective population size, leading to increased inbreeding and a lower fitness, in turn causing a lower effective population size, is known as the extinction vortex, Figure 1 (Frankham, 2005; Biere et al., 2012).

This means there is a theoretical threshold value for effective population size, after which the population enters this extinction vortex, leading to the inevitable population collapse. However, scientific approach and results of studies on the relation between population size and genetic diversity vary greatly between studies and species (Rubidge et al., 2012; Díez-del-Molino et al., 2018; Genereux et al., 2020). Due to the high environmental impact humans have, it is necessary for species to adapt to new or changing environmental conditions, or at least have the ability to do so in the (near) future. However, because smaller populations experience lower genetic diversity, this can lead to an inability to adapt to changing environments (Ouborg et al., 1991; Bijlsma & Loeschcke, 2012; Scott et al., 2020). It has been shown that populations with reduced genetic diversity often experience reduced growth and increased extinction rates, most likely as a result of inbreeding, causing harmful mutations to occur in a homozygous state. A decreased population size can increase the realized genetic load (fixation of deleterious genes in a population), leading to an overall decrease in a population’s fitness (Spielman et al., 2004; Díez-del-Molino et al., 2018; Ralls et al., 2020; Bertorelle 2022). Interestingly, not all inbred populations experience inbreeding depression to the same degree (Garciá-Dorado 2003), suggesting that homozygosity of specific variants cause the negative effects during inbreeding (Charlesworth & Willis, 2009). Inbreeding depression has largely been attributed to the accumulation of recessive harmful mutations in the genome: inbreeding increases the probability of these mutations to become homozygous and thus expressed (Ohta, 1973; Charlesworth & Willis, 2009; Agrawal & Whitlock, 2012). The key concept here is that while harmful mutations generally have a small fitness effect in heterozygous state (masked), in homozygous state they cause heritable diseases (realized). The potential negative impact that inbreeding will have on health and reproduction compared to an outbred population is referred to as “*genetic load*” (Dobzhansky, 1957; Kimura et al., 1963) which is mainly caused by expression of homozygous harmful mutations (Lynch et al., 1995; Garciá-Dorado, 2003; Allendorf et al., 2013). Understanding and predicting the factors causing fitness decline will aid to avoid a high genetic load in the future (Garciá-Dorado, 2012; Kyriazis et al., 2020; Bertorelle et al., 2022).

Genetics thus plays a crucial role in fitness of small populations, but nowadays genetic parameters are barely used in conservation of endangered species. The genetic status of a species is often overlooked when assessing conservation status, an alarming development according to many population geneticists (Laikre et al., 2020). It may therefore not be surprising that genome erosion parameters are currently a poor predictor of the conservation status in endangered species (Díez-del-Molino et al., 2018). We urgently need better predictors of populations under threat to be able to preserve and prioritize biodiversity in decline (Nogués-Bravo et al., 2018). Implementing genomics can play an important role in such predictors (Breed et al., 2019; Segelbacher et al., 2021; Willi et al., 2022).

### 1.2 The challenge of using genomics for conservation

While key concepts in conservation genetics were established decades ago, re-sequencing techniques enabled the study of complex population structure, local genetic signatures in the genome and the effect of specific mutations (Willi & Hoffmann, 2009). The availability of complete genomes of a range of species has opened up a treasure-trove of information for conservation biologists. Recent advances in genome sequencing techniques now enable genomic analysis of non-model species (Ekblom and Galindo 2010). Using tools originally developed for human and model species such as laboratory animals and livestock, we can unlock a wealth of valuable data about the population history and current genetic status of a species (Primmer, 2009; Ouborg et al., 2010; Segelbacher et al., 2021). Conservation genomics has huge potential, but up to now it has not been truly adopted by the conservation community (Shafer et al., 2015). Many questions remain to be answered and some applications have not sufficiently proven its value in the field (Garciá-Dorado, 2003; Breed et al., 2019). Despite the challenging process of translating of genomic tools into conservation, exciting opportunities are emerging (Segelbacher et al., 2021; Willi et al., 2022). Genomics assisted breeding for maintaining variation and reducing genetic defects is widely adopted in domesticated animals (Windig et al., 2004; Windig & Doekes, 2018), as well as genomics techniques to map recessive defects (Charlier 2008) and pinpoint recessive lethals have proven valuable in cattle (Charlier 2008, 2016) and pigs (Derks 2019). Such approaches can be adopted for endangered species as well (Bosse et al., 2015; Bosse et al., 2018). Genomics can provide valuable input for further genomics-assisted conservation efforts (Amos & Balmford, 2001; Shafer et al., 2015; Hohenlohe et al., 2020). However, a strong fundamental scientific basis is crucial for long-term success.

Understanding how genetic variation affects fitness differences between individuals lies at the heart of conservation genomic studies. This has become an achievable objective, now that the new sequencing technology enables the characterization of the full spectrum of variation in genomes. For conservation purposes, it is necessary to research the exact link between the genetic measures that can be taken and the survival chances of a population. The three main measures to consider are overall genetic diversity, recent inbreeding (autozygosity) and inbreeding depression caused by accumulation of deleterious genes. Increasing the genetic diversity of a population is essential to maintain the ability to adapt to changing environments, since genetic heterozygosity is essential for recombination and evolution to occur (Fisher, 1930). Inbreeding has major impact on the genetic fitness of a population, since it both increases the overall homozygosity of the genome, as well as driving rare deleterious mutations more often to the homozygous state than expected according to expectations under Hardy-Weinberg Equilibrium. Inbreeding depression has a large biological impact on wild populations, but can potentially be combatted relatively well, by crossing individuals suffering from the fixation of harmful (recessive) alleles with individuals that do not (Charlesworth, 1987; Lynch et al., 1995; Crnokrak & Roff, 1999). This can help remove the harmful allele variants from the population and increase genetic fitness. The genetic load of a population can dramatically increase when population size decreases. The genetic load is the amount of genes that are less than the maximal fitness genes, like deleterious recessive genes, which can get fixated in a population. By genetic screening and mating programs, these mutations can be bred out of a population, increasing the overall genetic fitness (van Straalen & Timmermans, 2002).

A population with low genetic diversity can be “genetically rescued” by crossing it with a different population, masking the effect of fixed harmful mutations (Keller & Waller, 2002; Tallmon et al., 2004; Edmands, 2007). This shows the potential of using genetic data for conservation efforts.

## 2 The quantitative determination of genetic erosion

Quantifying genetic erosion can be used as an indicator for viable population size and extinction risk. Especially when used temporally, this could offer conservationists a quantifiable measure to assess at risk populations (Reed & Frankham, 2003; Frankham, 2005; Díez-del-Molino et al., 2018; Leroy et al., 2018). This can be done by using old samples, like museum specimens, as a genetic “baseline” and comparing current genetic fitness to this baseline (museomics), even making use of DNA from extinct lineages (Díez-del-Molino et al., 2018). Using this methodology, the current population can be compared to pre-decline genetic diversity indicators, providing insight in effective population size and extinction risk (Gauthier et al., 2020). Such temporal comparisons are most effective when using genetic material from closely related lineages, as shown in gorilla (van der Valk et al., 2021), kākāpō (Dussex et al., 2021), and rhinoceros (Liu et al., 2021).

In order to assess the extinction risk status, a standardized unit needs to be implemented in order to link a certain amount of genetic erosion to risk of extinction. Genomic data holds potential to reflect past and current effective population size, as well as masked and realized genetic load. Clear threshold values of effective population size and risk of extinction, derived from genomic data, could serve as indicator for IUCN red list assessment (Garner et al., 2020). Threshold values can be based on minimum viable population size (MVP) estimates, like the 50/500 rule or other, estimated or calculated MVP values (Franklin, 1980; Jamieson et al., 2012; Reed et al., 2003; Rosenfeld, 2014; Ryman et al., 2019; Traill et al., 2007; Wang et al., 2019). Franklin’s 50/500 rule is based on estimates for short term inbreeding effects, based on the amount of inbreeding accepted by animal breeders (N_e_ = 50) and long term effects based on theoretical prevention of genetic drift and massive loss of gene variance (N_e_ = 500). This reasoning has proven extremely important for the conservation community, however a genomics-informed equivalent to these rules would be preferred in conservation efforts, adopting quantified methods from sequence data, to help indicate at risk species.

Providing conservationists with a quantifiable measure for genetic erosion to assess species at risk of extinction would be very helpful in conservation efforts. In order to use genetic erosion measurements in the assessment of at risk species, the link between a declining population and the presence of genetic erosion needs to be established first.

Ideally, all of the genetic erosion measures are taken into account to calculate an effective population size. The effective population size can then be used to assess the extinction risk of a species. By using a measure like the 50/500 rule, as proposed by Franklin, tiers can be created to give a certain genetic erosion score. This erosion score is in the form of effective population size, which corresponds to a certain extinction risk and specific measures that can be taken. However, in order to do this, the 50/500 rule for MVP is in need of revising (Franklin, 1980; Frankham et al., 2014; Rosenfeld, 2014; Steeves et al., 2017). Although the original determination of 50/500 is simple and effective, it is not species/clade specific and based on vastly inferior data to what is currently available with modern genetic techniques. Implementation of a clade or class specific MVP size could, for instance, better deal with traits like kin recognition and litter size and class specific gene variance and mutation rates. Consensus on a MVP that includes genomic factors like diversity and genetic load is required to build up this “conservation guideline” and the effective population size needs to be comprised of several quantitative genetic erosion markers. Here we discuss three quantitative genetic erosion indicators that could bring this field forward.

### 2.1 Overall genetic diversity

#### 2.1.1 Concept

Overall genetic diversity is essential for the survival of a species. Genetic diversity is recognized as one of three important levels of biological diversity by the Concention on Biological Diversity (CBD; O’Brien et al., 2022). Genetic diversity is the fuel for populations to evolve and adapt to changing environments (Frankham, 1996; Reed & Frankham, 2003; Hughes et al., 2008). Whether the conservation focus should be on loci of adaptive potential, or overall genomic diversity is still debated (see Des Roches et al., 2021; DeWoody et al., 2021; Teixeira et al., 2021). Genetic erosion of the overall genetic diversity is characterized by reduced heterozygosity at the individual level, or reduced pi at the population level. Loss of genetic diversity can have many different causes, like habitat fragmentation, the founder effect or population bottle necks (Gallardo et al., 1995; Keller & Taylor, 2008; Lowe et al., 2005; Reed & Frankham, 2003; Schlaepfer et al., 2018). However, all these causes are directly related to effective population size (*N*_e_). Although the concept of effective population size is rather intuitive, and extremely important for conservation genomics, its exact definition is can be less valuable from a practical point of view (Waples 2022). Therefore, it is suggested to view *N*_e_ as the determinant factor for the rate of random genetic drift across the entire genome in the offspring generation, and in that capacity *N*_e_ is associated with inbreeding and genome-wide heterozygosity.

**FIGURE 2:**
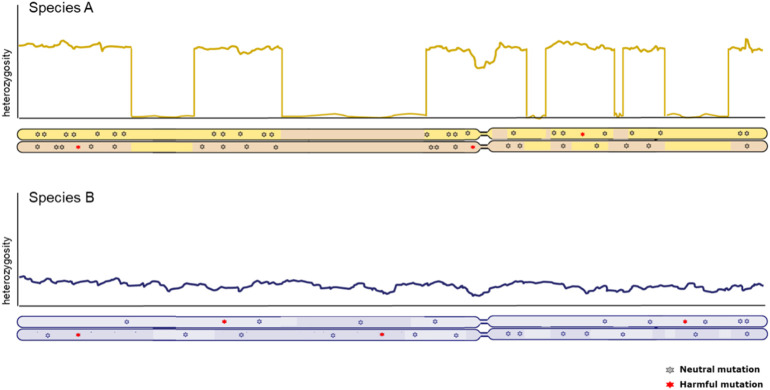
Chromosome-wide distribution of heterozygosity in individuals from two distinct populations with equal genome-wide levels of diversity. *X*-axis represents the full length of a chromosome, with the two copies illustrated below. *Y*-axis represents the heterozygosity level derived from the number of SNPs between both copies of the genome in an individual. The long stretches without heterozygosity due to identical copies of the same segment in the top individual are know as Runs of Homozygosity (ROH).

Genome-wide patterns of heterozygosity within the genome of one individual provide crucial information about the genetic status of populations. Inferences of historic effective population sizes are a popular feature in the description of population history (Li & Durbin, 2011) and strength and duration of population bottlenecks convey cues about potential loss of adaptive capacity as well as the potential for purging of harmful mutations (Bertorelle et al., 2022). However, equal genome-wide levels of genetic diversity are not necessarily indicative of the same conservation concern (illustrated in Figure 2). A high background level of diversity can be interrupted with long runs of homozygosity, indicative of more recent inbreeding. Equally distributed genome-wide heterozygosity points at more stable effective population size, but smaller (Ceballos et al., 2018a). Therefore, how diversity is distributed along the genome is an important factor to consider. Naturally, caution should be taken when interpreting heterozygosity levels and connecting these to effective population size; one should be aware of underlying assumptions of panmixia and migration, which can distort the relationship between heterozygosity and effective population size.

#### 2.1.2 Methodology

Quantification of overall genetic diversity has traditionally been done using an array of methods, including SNP analysis from both whole genome sequencing data and SNP-chips, but also allelic heterozygosity determination and even pedigree data (Hughes et al., 2008). Although SNPs may not be as directly indicative of adaptive potential as allelic heterozygosity, both are indicative of an organism’s ability to adapt. A lower overall genetic heterozygosity is therefore indicative of a small past and/or contemporary effective population size and a lesser adaptive potential. When multiple samples per population can be obtained, this would allow for the estimation of not only heterozygosity on a single-SNP basis, but also haplotype richness which is a reflection of the number of segregating haplotypes in a population at a given locus (i.e., López-Cortegano et al., 2019). For such estimates to be reliable, multiple individuals need to be sampled and phasing should be accurate, which remains challenging for many wild/endangered populations. The Watterson estimator is a method to describe the genetic diversity in a given population, in which the genetic diversity is a function of effective population size and per generation mutation rate (Watterson, 1975). When using overall genetic diversity as a marker for genetic erosion, the use of the Watterson estimator does not fully suffice. By using more extensive sampling (>4 individuals) per popoulation, better estimates can be achieved, especially for more recent *N*_e_ by incorporating the observed spectrum of linkage disequilibrium (Santiago et al., 2020). For single (diploid) individuals, however, patterns of heterozygosity form an acceptable proxy.

This can be done temporally, allowing for a comparison with, for instance, museum specimens. If there are no specimens available, this technique can also be used on a single sample and non-temporally (Luikart et al., 2010; Wang et al., 2016).

Ideally, a quantification of heterozygosity would be made using the entire genome sequence of several individuals, since this gives the highest accuracy. However, the amount of high quality DNA samples that can be acquired for endangered species is often very low and whole genome sequencing is still considered expensive. Besides the practical issues of whole genome sequencing, one can argue that this method yields an overkill of information for conservation purposes (Allendorf et al., 2010). High-density SNP chips provide an accurate enough and much cheaper alternative. A single full genome is needed to screen for SNPs and minor allele frequency (MAF) locations for the SNP chip design (Groenen et al., 2011; Cheng et al., 2016). After the SNP chip is developed, individuals of the species can be checked for overall homozygosity of the genome in a relatively cheap and fast manner. The use of high-density SNP chips also allows for identification of loci that are genetically eroded (Allendorf et al., 2010). However, the development of SNP chips is only feasible if the chip will be used regularly. Affordable alternatives have been developed for discovering SNPs that have as an advantage their lower costs (Du et al., 2012). These strategies include the reduction of genome complexity by digesting the genome with one or more restriction enzymes, after which a selection of small fragments is taken and sequenced by high-throughput sequencing. After this, fragments are aligned to either a reference genome or assembled. Thus, only a small fraction of the genome of many individuals is sampled (Nielsen et al., 2011; Greminger et al., 2014; Perea et al., 2016). We highlight that if the density is sufficient to disentangle homozygous and heterozygous segments, such reduced representation sequencing strategies will fulfill its purpose to inform conservationists about the distribution of diversity along the genome, which is indicative of past and recent bottlenecks.

### 2.2 Runs of homozygosity

#### 2.2.1 Concept

Runs of homozygosity (ROH) are long continuous tracts of consecutive homozygous single nucleotide polymorphisms (SNPs) (Gibson et al., 2006). These homozygous segments of DNA are a result of autozygosity, when parents with a common ancestor reproduce and pass on the identical genomic segment to their offspring. This causes shared chromosomal segments to be passed on to their offspring, with little to no genetic diversity (Figure 2) (Peripolli et al., 2017). ROH are conceptually similar to Identical-by-descent (IBD) tracts between individuals, but captured in one individual. Since most deleterious alleles in a population are at least partially recessive, an increase in homozygosity causes an increase in the homozygosity of a deleterious allele, causing inbreeding depression (Charlesworth, 1987). If this occurs in a small population and carriers of homozygous deleterious alleles reproduce, inbreeding depression can impact both individual as well as population fitness. Inbreeding depression in bird and mammal populations are shown to significantly affect birth weight, survival, reproduction, resistance to disease, predation and environmental stress (Keller & Waller, 2002). The level of ROH correlates with the level of inbreeding of an individual, thus potentially leading to inbreeding depression. Since inbreeding depression causes fitness decline and loss of genetic variation, this is an important measure for genetic erosion (Hedrick et al., 2000; Bijlsma & Loeschcke, 2012). ROH are increasingly used to measure autozygosity and inbreeding from genomes, and indeed can be correlated with fitness reduction (Stoffel et al., 2021). Some evidence has emerged that long runs of homozygosity tend to contain more homozygous harmful mutations (Szpiech et al., 2013; Bosse et al., 2018; Bortoluzzi et al., 2020). However, the effect of the age of the ROH and the traits affected by inbreeding depression differ greatly in livestock (Zhang et al., 2015a; Doekes et al., 2021), demonstrating that not all inbreeding is depressing; especially “young” ROH tend to result in inbreeding depression, since low-frequency recessive variants are exposed for the first time, whereas ROH stemming from haplotypes that have been homozygous previously are less likely to carry highly detrimental mutations because of purging.

#### 2.2.2 Methodology

The resulting homozygous segments can be detected using various data types, as long as the density is sufficient to separate heterozygous from homozygous stretches. SNP chips are a valuable and easy source to acquire genetic data for ROH analysis (Allendorf et al., 2010; Zhang et al., 2015b; Rodríguez-Ramilo et al., 2015; Bosse et al., 2018; Islam et al., 2019). The data produced can be analyzed for homozygous regions and gives an accurate quantification of the amount of autozygosity (McQuillan et al., 2008; Keller et al., 2011; Ferenčaković et al., 2013). However, the use of SNP chips is only possible for species with well-established SNP chips, and when applying chips designed for other species, it can lead to ascertainment bias and an overestimation of ROH coverage (Bosse et al., 2012).

An essential requirement for the use of SNP chips is a standardized method of SNP data collection and processing. Peripolli and colleagues showed significant differences between different analytical tools used to assess SNP data for ROH frequencies (Peripolli et al., 2017). Many factors in the analytical analyses of SNP data influence the results, therefore guidelines need to be made about what type of tools to use and in what settings. This is especially important for the analysis of ROHs in SNP data, since the results are influenced greatly by parameter settings, like how many heterozygous positions are allowed in an ROH and in minimal ROH length (Howrigan et al., 2011; Ku et al., 2011).

The use of ROH as a quantifiable measure of genetic erosion poses some issues however. For instance because the levels of ROH are not always caused by inbreeding, but could also be the result of strong directional selection on a desired variant, likely increasing fitness instead! Also, the fact that levels of ROH differ according to their position on the chromosome may pose some difficulties to implement genomic ROH coverage. However, the main challenges with the use of ROH levels to quantify genetic erosion lie within the derivation of ROH itself. Since there is no consensus within the scientific community on the definition of ROH, the quantification of ROH can differ between studies (Meyermans et al., 2020). Besides a lack of consensus on what is defined as ROH, like cut-offs for what size of homozygous elements are considered ROH, the derivation of the results also lacks consensus. Research by Ceballos et al. (2018b) concluded (low coverage) whole genome sequence data to produce vastly different amounts of ROH than SNP array data. SNP array data mainly missed the shorter ROH lengths. Peripolli et al. (2017) even found three ROH analysis algorithms to give significantly (*p* < 0.001) different ROH frequencies. Across mammals, trophic level, body mass, and latitude have significant effects on *θ* and ROH burden (Brüniche-Olsen et al., 2018). These examples indicate the necessity for consensus on all parameters in ROH determination, at least when performing museomics or when comparing animals within the same taxa. Definitive choices on what length of homozygosity is considered a ROH, which analysis tool is used, minimal SNP chip density and optimal genotyping error is needed (Meyermans et al., 2020). A reasonable working definition is the fROH, the proportion of the genome covered by the sum of homozygous segments. Some caution may be taken when inferring inbreeding directly from homozygous genomic segments using a cut-off approach all together; alternative approaches use Hidden-Markov-Models to infer the distribution of homozygous segments in genomes (Narasimhan et al., 2016), with the advantage that Homozygosity-by-descent (HBD) segment length is believed to reflect inbreeding age, and therefore provides additional information on population history (Bertrand et al., 2019; Druet and Gautier, 2022).

### 2.3 Genetic load

#### 2.3.1 Concept

The prevalence of deleterious mutations, also known as genetic load, can decrease individual fitness, especially when they occur in a homozygous state (Blomqvist et al., 2010; Bertorelle et al., 2022). However, the effects of deleterious mutations on a population level is usually low. This is, until population size decreases, causing it to suffer from inbreeding. This can cause the (recessive) deleterious mutations to become homozygous, causing a decreased fitness and further leading an already diminishing population into the extinction vortex (Higgins & Lynch, 2001; Fagan & Holmes, 2006; Agrawal & Whitlock, 2012).

Since the accumulation of deleterious gene variants can contribute to the downfall of an entire population, this is an important factor of genetic erosion to take into account. When natural selection is unable to remove harmful mutations, the genomes of such populations will accumulate harmful mutations, potentially leading to extinction. In small populations, chance plays a bigger role in the fate of a mutation and slightly deleterious mutations may accumulate with higher probability, causing a more rapid population fitness decline (Keller and Waller 2002; Charlesworth and Wills 2009). This phenomenon is most apparent in obligate asexual organisms (Muller’s ratchet: Muller, 1964; Gabriel et al., 1993). The quantification of the genetic load in a population is rather difficult, since most techniques ask for phenotypic data. However, when used temporally, genome sequence data offers the possibility to compare old, pre-population decline, samples to current population genetics.

#### 2.3.2 Methodology

Understanding how genetic variation affects fitness differences between individuals lies at the heart of conservation genomic studies. This has become an achievable objective, now that the new sequencing technology enables the characterization of the full spectrum of variation in genomes of multiple individuals: 1) unconditionally deleterious variants, and 2) structural variants that create genetic incompatibility when outcrossed. Long-read and high throughput sequencing technologies now open up exciting possibilities to actually pinpoint potentially harmful mutations and structural variants in individual genomes (Li et al., 2010; Henn et al., 2015). The deleteriousness of a variant can be predicted bioinformatically, based on its effect on gene functioning (protein changing, stop-gain) and the degree of conservation of the sequence across species (Cooper et al., 2005; Kumar et al., 2009; Wang et al., 2010). These techniques have recently been applied to estimate genetic load in human populations and domesticated species. The implementation of multiple annotations, such as specific gene function or regulatory elements have proven successful for in-silico predictions of the effect of disease causing mutations in human (Kircher et al., 2014). Although multiple factors contribute to inbreeding depression, quantifying the harmful mutations from single genomes is an important first step towards the implementation of genetic load into conservation programmes (Bertorelle et al., 2022).

Temporal use of genomic data offers the possibility to monitor loss-of-function (LoF) variants and synonymous gene variants. LoF variants, like stop-gain, splice site, stop-loss and frame-shift mutations can be uncovered using genetic techniques and represent part of the genetic load in an individual. Although not all LoF have negative fitness effects, it is assumed that an increase in such mutations likely contribute to the lowered fitness of an individual. The quantification of LoF mutations can serve as a good proxy for genetic load quantification and is likely the best method we currently have (Pagel et al., 2017). By looking at the ratio of LoF variants to synonymous gene variants, this gives an indication of the increase of genetic load within a population over time (Lynch et al., 1995; Díez-del-Molino et al., 2018).

Knowledge on the temporal increase in genetic load is indicative of the overall genetic erosion in a population. The theoretical increase in genetic load in a diminishing population has also been empirically tested and found to uphold this hypothesis (e.g., Lohr & Haag, 2015; Liu et al., 2020). It is therefore found to be a good indicator of genetic erosion, but, to the knowledge of the author, it is not yet possible to directly link this to an effective population size. It does, however, indicate genetic erosion and is indicative of how at-risk a population is. Lynch et al. (1995) reported on the vulnerability to extinction of a population with effective population sizes lower than 100 individuals, due to the genetic load. Both the beforementioned vulnerability and the known increase in effects of genetic load when inbreeding increases make it an important factor to take in account when assessing genetic erosion and at risk populations.

A very apparent example of genetic load is hybrid load. Hybrid load is the effect of hybridization of closely related species, with offspring with lower fitness than both parents. Either the combination of species-specific genes can lead to a deleterious phenotype, or the resulting mixed phenotype is of a lower fitness than either parent species. The effect of natural and anthropogenic hybridization has been shown in several fish, but overall effects of hybridization events vary from increased fitness to non-viable offspring (Muhlfeld et al., 2009; Chafin et al., 2019). A decreased fitness due to hybridization can also be considered genetic erosion. Besides a decreased fitness, hybridization also greatly impacts the authenticity of a species or population.

## 3 Practical examples and recommendations

### 3.1 Genetic erosion in wild populations

Genetic erosion has so far been concluded for species in several classes of animals: mammals, fish, amphibians, insects and birds (e.g., Hutchinson et al., 2003; Noël et al., 2010; Turlure et al., 2014; Quintinilla et al., 2015; Potvin et al., 2017; Mathur et al., 2019; Thompson et al., 2019; Gauthier et al., 2020; Van der Valk et al., 2021). It seems, that the techniques currently available to assess and quantify genetic erosion are welcomed by the community. However, current literature suffers from two major shortcomings: A reluctance to conclude genetic erosion, and a major sampling bias.

Although genetic erosion has been concluded for several different classes of animals, the same goes for a lack of genetic erosion (Martinez-Cruz et al., 2007; Ugelvig et al., 2011; Reiniers et al., 2014; Dussex et al., 2019; Van der Valk et al., 2021). However, the question is whether there is truly a lack of genetic erosion or a reluctancy to conclude genetic erosion. Would the same conclusion be drawn, were different evaluation techniques used, or different standards taken as to what is considered genetic erosion, and what is not? Many papers describe features of genetic erosion, but conclude genetic erosion to be “low” or “inconclusive.” This highlights the demand for clear (quantitive) guidelines to what is considered genetic erosion. Since no consensus exists on the minimum quality of genetic data, assessment techniques and threshold values used in the evaluation of genetic erosion, concluding genetic erosion is arbitrary and authors are reluctant to do so.

The relationship between population decline or habitat fragmentation as reflected in genome erosion, and IUCN conservation status is far from clear (Brüniche-Olsen et al., 2018; Díez-del-Molino et al., 2018; Garner et al., 2020). This can partly be assigned to sampling bias in conservation genetics studies. Firstly, research into endangered species is given priority over non-endangered species or populations. This skews the data, as hardly any data exists on healthy populations and non-threatened species. Besides that, most papers that look for genetic erosion find it (see Supplementary Table S1). This might be due to the fact that most researched populations are those that are threatened by population decline or habitat fragmentation, or because threshold values are arbitrary and not based on empirical evidence, based on baseline overall heterogeneity, ROH and genetic load of non-threatened populations. However, some trends suggest that indeed endangered species have more eroded genomes, especially when related taxa are considered (Zoonomia Consortium 2020).

### 3.2 Quantified genetic erosion data

The complexity of genetic erosion and the factors influencing it ask for a robust standardized method, that can be confidently used for conservation purposes. All three different quantification methods, discussed before, each with their own benefits and limitations should be included, to deal with the complexity of genetic erosion. For example, a population that has been small for a long time has high potential to become extinct due to a lack of overall heterozygosity and therefore a lack of adaptive potential. However, this population might not show a lot of inbreeding or high levels of deleterious mutations, due to high selection pressure, purging strongly deleterious recessive alleles (García-Dorado, 2012; Robinson et al., 2018; van der Valk et al., 2021). Another example of the complex nature of genetic erosion is the purging of deleterious mutations due to heavy inbreeding or severe bottlenecks (Glémin, 2003; Grossen et al., 2020).

Ideally, generalized methods should include the effective population size, calculated from both the ROH and the overall heterozygosity. This allows the inference of the past as well as current population size. Since genetic load can not be linked to effective population size, it is currently hard to incorporate this in the quantification of genetic erosion and at risk populations in the same manner as overall heterozygosity and inbreeding. However, it is a strong indicator of genetic erosion and should not be left out, especially since the effects of inbreeding depression are increased by higher genetic load. For now, it can therefore be used as an additional indicator of genetic erosion. However, with our knowledge about evolution and trajectories of harmful mutations in populations, we obtain increasing understanding of the fate of such alleles in populations, given their demographic history (Bertorelle et al., 2022). So even though genetic load cannot be measured directly, by simulating genetic load based on sequence-derived information about past demographic events, we can estimate the level of masked and realized load in populations.

The difficulty of incorporating genetic load is the determination of recessive deleterious alleles, which is currently hampered by technological limitations. In pioneer studies the method to determine whether deleterious mutations are accumulating in a population is by collecting phenotypic data and linking it to specific alleles. This labor intensive process is not realistic for every population. A more realistic method is to establish a system of model species with well-established traits and their genetic markers. Model species can be used to validate the prediction of genetic load from the genome sequence. Once this purely sequence-based method is validated, it could be used for non-model species. Although this is a system that is not yet established, it would be an important indicator for at risk species. It allows the incorporation of genetic load in the risk assessment of species, without the need to collect phenotypic data for each species individually.

The importance of incorporating genetic load is most apparent in the prevention of deleterious gene variant accumulation, since these are the driving force behind inbreeding depression. However, despite the low abundance of lethal recessive alleles or hybridization events, their importance should not be underestimated. Deleterious mutations with high fitness impact, like recessive lethal mutations can cause the extension of a population bottleneck, or even the extinction of a species (Campbell, 2016). Therefore, a screening for lethal recessive alleles should be incorporated in the risk assessment of every species. A similar assessment of possible hybridization would have to be incorporated in the risk assessment of every species, since hybridization can greatly impact offspring fitness or even lead to the extinction of species (Allendorf et al., 2001; Muhlfeld et al., 2009). This screening is important for conservation, since it is fairly easy to purge these high impact lethal recessive genes from the gene pool in the short term, using breeding programs to decrease the allele’s frequency in the population. In future risk assessment, outbreeding depression, the effect of mixing two long-time separated populations of a single species leading to lower genetic fitness, can also be incorporated.

### 3.3 Genetic rescue and hybridization

**FIGURE 3:**
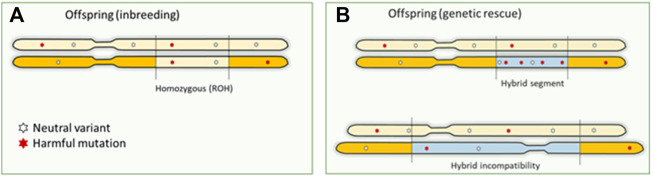
Genomic consequences of inbreeding and outbreeding. **(A)**. Offspring inherit identical DNA twice when parents are related, which means that harmful recessive mutations become homozygous and expressed. This is the primary mechanism causing inbreeding depression. **(B)**. Outbreeding (genetic rescue) introduces DNA from another source into a population, increasing genetic variation. However, this can also increase the number of harmful mutations as a masked load. These mutations could become expressed in homozygotes in future generations, as inbreeding continues to convert the masked load into a realized load. Also, large structural differences between the donor and source will result in hybrid incompatibilities.

A possible solution to counteract the effects of inbreeding depression is to introduce genetic material from another population. Introduced genetic variation can neutralize inbreeding depression effects by making loci with recessive harmful mutations heterozygous (Hedrick & García-Dorado, 2016; Fitzpatrick et al., 2020). The resulting increase in fitness is referred to as “genetic rescue,” and it effectively transforms the realized load into a masked load (Whiteley et al., 2015; Bertorelle et al., 2022). Some well-known success stories of genetic rescue entail the Florida panther, and the American bison (Hedrick, 2009; Johnson et al., 2010; Hostetler et al., 2012). Typically, large mammalian species are not a genetically homogeneous group, and may consist of different subspecies, populations and sub-populations. Unfortunately, (sub)species are lost without even been properly described. These groups can be on different evolutionary (and potentially adaptive) trajectories and mixing may lead to outbreeding depression (Hedrick & García-Dorado, 2016). Introgression and hybridization can also lead to genomic incompatibility, with as a special case the mito-nuclear incompatibility in the so called “mothers curse.” Purely maternal inheritance of mitochondrial DNA disables selection against harmful mutations in the males (Gemmell et al., 2004), resulting in mtDNA contributing to male fitness reduction, for example reduction in male lifespan in human (Milot et al., 2017). Concerns about outbreeding depression hinder the potential of human-mediated gene flow (Frankham et al., 2011). Genetic rescue poses three possible “genomic problems”: 1) if the introduced DNA contains unconditionally harmful mutations, the total genetic load may actually increase concurrently with genetic variation (Edmands, 2007; Frankham et al., 2011) (exemplified in Figure 3). The first generation of hybrids could still express increased fitness, as the load is masked in heterozygote genotypes. However, once the immigrant DNA becomes homozygous in future generations, the advantage may be lost, and novel genetic defects may arise (Hedrick & García-Dorado, 2016). 2) Introduction of variants that are harmful to the recipient population (e.g., maladapted variants) and structural variants that cause genetic incompatibilities. Outbreeding depression because of genomic incompatibilities caused by large structural variants or maladaptation may occur, but its role of structural variation in inbreeding and outbreeding depression has been largely overlooked (Hoffmann et al., 2020). Structural variants can have pronounced phenotypic impact–they can lead to genomic incompatibilities in the first generation of hybrids or disrupting gene functioning and regulation of modifying gene dosage in further generations. Multiple studies have highlighted their role in functional changes across populations and species (Mahmoud et al., 2019). 3) The benefits are transient as the total genetic load is not necessarily reduced by genetic rescue. On the other hand, if the groups are too small without migration between the groups this may lead to high inbreeding rates within the groups and associated loss of fitness. Genomics can provide important cues on whether different Evolutionary Significant Units (ESUs) exist that warrant independent conservation management. General predictions about the success of genetic rescue have proven difficult so far (Verhoeven et al., 2010; Frankham et al., 2011; Hedrick et al., 2014; Frankham 2015). Experimental crosses between inbred lines have demonstrated the potential benefits as well as risks of genetic rescue in *Drosophila*, but these studies did not quantify harmful mutations in their experimental populations (Bijlsma et al., 2010). Knowledge about harmful mutations in the donor population is therefore crucial for predicting the success of genetic rescue (Frankham et al., 2011; Whiteley et al., 2015; Kyriazis et al., 2020). However, pinpointing which mutations contribute to genetic load continues to be challenging in non-model organisms (Bertorelle et al., 2022).

The distribution of segments with particularly high- or low levels of heterozygosity provide further insights into more recent demographic events.

**FIGURE 4:**
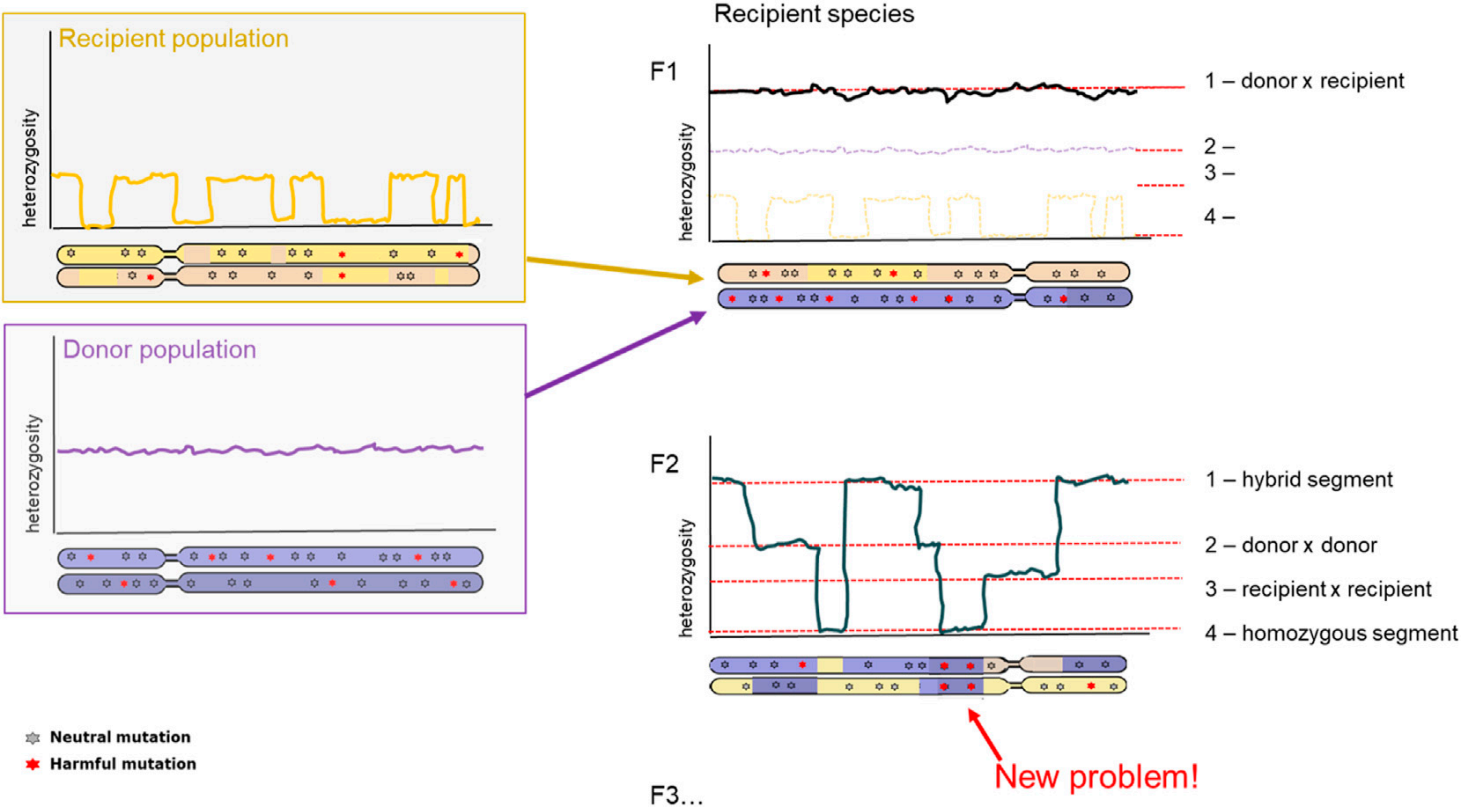
Diversity and genetic load after hybridization. Representation of chromosome-wide heterozygosity in an individual, sampled from the Donor population (top, purple); Recipient population (bottom, orange); and the first generation hybrid offspring (right panel). First generation hybrid (F1; top panel) and second generation hybrid (F2, bottom panel) are depicted. Genetic variants are indicated as grey (neutral) and red (harmful).

A clear consequence after hybridization is outbreeding depression. Outbreeding depression is the consequence arising from mixing two separate populations in an attempt to increase genetic variation. However, since the two populations have become too different over time, they have poorly compatible genome sequences leading to lower fitness offspring. The resulting offspring can experience lower fitness through different mechanisms: intrinsic and extrinsic outbreeding depression. Intrinsic outbreeding depression can occur from genic or chromosomal incompatibilities, whereas extrinsic outbreeding depression can occur from reduced adaptation to local environmental conditions. Intrinsic outbreeding depression causes a decrease in fitness when the resulting offspring suffers from a disruption in intrinsic interactions between genes. Extrinsic outbreeding depression causes the offspring to be adapted to neither of the local environmental conditions (Edmands, 2007; Allendorf et al., 2010). A third mechanism behind outbreeding depression is an increase of genetic load. Increase in genetic load can be a primary determinant of extinction risk in future generations after introduction, therefore development of methodology to determine such load-causing mutations is essential (Kyriazis et al., 2020; Willi et al., 2022). For isolated populations, first should be evaluated whether inbreeding depression is likely, quantifying the masked load (cf. Inbreeding load) in populations. If the masked load is low, care should be taken to not inadvertently increase this *via* genetic rescue, even though long-term adaptability is increased with higher levels of genetic variation (Figure 4).

The incorporation of outbreeding depression in the genomic estimation of a species genetic fitness is an important incorporation for two reasons. First of all, it allows conservationists to identify populations already suffering from outbreeding depression and take this into account when assessing at risk populations. This could be useful for populations with decreasing habitat, due to anthropogenic influence or climate change, causing populations that have been separated for a long time to mix. The second reason is to prevent anthropogenically induced outbreeding. For conservation purposes it might seem to make sense to introduce new genes into the gene pool, but by monitoring what genes are introduced and assessing their compatibility, outbreeding depression can be prevented.

## 4 Future prospects

### 4.1 Early warning system based on genetic load

Overall, there does seem to be a strong indication that genetic erosion occurs in declining populations. It is, however, only observed when the decline is not extremely fast, since it would lead to extinction even before the first genetic effects establish in the genome. When a species has not experienced a very small, but stable, overall population for a very long time, it can also lack the genetic indicators for erosion, since these could be purged from the population (Bertorelle et al., 2022). These are, however, important factors to take into account when using these genetic factors in conservation. The use of genetic erosion markers in conservation could serve a great purpose in declining populations, as a signature of the current state of a population, its adaptive potential and a warning system for future population decline. By using accurate measures from sequence data to predict the masked load, we will be able to measure the genetic load within their populations before genetic defects become apparent. Managers of wild and captive populations can measure genetic load without having to capture and breed animals to evaluate the effect of inbreeding and bad mutations on fitness, thereby providing them with an early warning system. Furthermore, by integrating information on current genetic load into breeding programs, negative effects of these mutations may be directly reduced by breeding for “lower genetic load” next to managing the inbreeding rate in breeding programs.

### 4.2 The promise of related genomics fields for conservation genomics

Thus far, the role of structural variation in inbreeding and outbreeding depression has been largely overlooked (Hoffmann et al., 2020). The fast developing field of pan-genomics could fill this gap and lead to the implementation of structural variation in conservation genomics. Pan-genomes represent the genomic diversity of a species and includes core genes, found in all individuals, as well as variable genes, which are absent in some individuals (Golicz et al., 2020). Pangenomics are particularly useful to identify presence/absence variations (PAVs), copy number variations (CNVs), and other, miscellaneous variations (mostly large structural variants). The pangenomics concept has become widely adopted in plant breeding, and it is emerging in animal breeding (Bayer et al., 2020; Crysnanto & Pausch, 2020). By translating these concepts to endangered species, we can obtain the full breadth of diversity across subspecies, and obtain higher understanding of the evolution of gene families, gene losses and gains, and the structural variations within this species (Figure 3B). While an increasing number of whole genome sequence data has accumulated, and a huge number of single nucleotide variants (SNVs) has been identified, current approaches are still suffering two major shortcomings: 1) the use of a reference genome derived from a single (hybrid) haplotype and 2) the use of short read sequence technologies in genetic analyses. As a result, on average only 85%–90% of the genome of an individual is targeted and large structural variants are difficult or impossible to identify (Audano et al., 2019; Chaisson et al., 2019). Typically, these sequences are among the most variable in the genome and strongly contribute to genomic incompatibilities between populations and form reproductive barriers. Perturbation by structural variation of genic sequence as well as noncoding DNA regulatory elements and structural chromatin features, plays a major role in speciation and adaptation (Fudenberg & Pollard, 2019). More insight in structural differences that can now be obtained using long-read sequencing will lead to an expansion of Frankham’s reintroduction framework by including SVs instead of only karyotypic differences (Frankham et al., 2011; Frankham, 2015), by predicting genomic incompatibilities and associated fitness loss and potential reproductive barriers. Finally, the potential of the “sister field” of quantitative genetics has huge potential to better understand genomic architecture and genetic variation underlying complex quantitative traits that are important for evolutionary change (Gienap et al., 2017; Ørsted et al., 2019; Capblancq et al., 2020; Teixeira et al., 2021; Willi et al., 2022). Further adoption of quantitative genetics methods, such as genomic prediction, could bring the field forward (Bonnet et al., 2022).

### 4.3 The need for consensus

An important issue pointed out by this review in the incorporation of genetic erosion in conservation is the shear complexity of genetic erosion, but the demand for simple measures by conservationists. One example of its complex nature is kin recognition. When inbreeding is used as a marker for genetic erosion and adaptive potential and a species avoids reproducing with kin, the amount of inbreeding is kept to a minimum and it might not be indicated as genetically endangered to conservationists. This has, for instance, been suggested in urban populations of red-backed salamanders (*Plethodon cinereus*) (Noël et al., 2010). The authors found no signs of inbreeding, even in populations in habitats as small as 0.5 ha. Genetic erosion could really help conservationists by providing another marker for a species’ performance, but the effectiveness may be class or species specific. Before the implementation of genetic erosion can be realized, the main necessity is gaining consensus, funding and bridging the gap between scientific research and the implementation in conservation. Scientists need to get to a consensus on which techniques need to be used, what threshold values need to be implemented and what new knowledge is required to realize this. All this is needed in order to provide conservationists with the information they need. To do this, funding needs to be allocated to the real world applications of recent technological advancements and communication between conservationists, geneticists and policy creators need to be improved (van Oosterhout, 2020). Recent efforts have made important contributions to bridge the gap between the vast scientific progress in the field and applicability (Hoban et al., 2020; O’Brien et al., 2022), leading to the much-needed incorporation of genetic data in the assessment of at risk species.
