# Supplementary material for: Challenges in quantifying genome erosion for conservation
Source: Front Genet. 2022 Sep 26;13:960958. doi: 10.3389/fgene.2022.960958 (PMC9549127; doi:10.3389/fgene.2022.960958)
Supplement: Supplementary file 1 [file Table1.pdf]

Supplementary Information 1

Table 1. Genetic erosion and conservation status of 49 wild species. Genetic erosion concluded by the authors is indicated. Temporal change is determined using the difference in genetic markers for genetic erosion of the declined population, compared to a pre- population decline sample of DNA. "Least Concern" (LC), "Near Threatened" (NT), "Vulnerable" (VU), "Endangered" (EN), "Critically Endangered" (CE) and "Extinct in the Wild" (EW) represent the different levels of conservation status. The presence of low overall heterozygosity, runs of homozygosity (ROH) and genetic load is given. The method indicates how the genetic data was obtained on which the analysis was performed.

Species	Genetic erosion	Conservation status	Heterozygosity	ROH/ Inbreeding	Genetic Load	Method	Reference	Comments
Channel island fox *(Urocyon littoralis)*	Yes	NT	X		X	WGS	Robinson et al., 2016	
Florida panther *(Puma concolor coryi)*	Yes	EN	X	X		WGS	Saremi et al., 2019; Uphyrkina et al., 2002	
Puma *(Puma concolor)*	No	LC				WGS	Saremi et al., 2019	
Grauer's gorilla *(Gorilla beringei graueri)*	Yes (temporal)	CE	X	X	X	WGS	Van der Valk et al., 2019	
Mountain gorilla *(Gorilla beringei beringei)*	No (temporal)	CE				WGS	Van der Valk et al., 2019	Continuous low population purges selectively bad gene variants very strongly
Humpback chub *(Gila cypha)*	Yes	EN			Hybrid load	ddRAD	Cha et al., 2019	
Bonytail *(Gila elegans)*	Yes	CE			Hybrid load	ddRAD	Cha et al., 2019	
Roundtail chub *(Gila robusta)*	Yes	VU			Hybrid load	ddRAD	Cha et al., 2019	
Black rhinoceros *(Diceros bicornis)*	Yes (temporal)	CE	X			MicroSattelite	Moodley et al., 2017	Introduction of new haplotype through outbreeding
Alpine chipmunk *(Tamias alpinus)*	Yes (temporal)	LC	X			MicroSattelite/ Targeted Exon Genotyping	Bi et al., 2019; Rubidge et al., 2012	Declining habitat due to climate change
Lodgepole chipmunk *(Tamias speciosis)*	No	LC				MicroSattelite	Bi et al., 2019; Rubidge et al., 2012	No habitat decline
Desert bighorn sheep *(Ovis canadensis)*	Yes	LC	X			MicroSattelite	Epps et al., 2006	Smaller populations (driven by climate change) show genetic erosion
Boreal woodland caribou *(Rangifer tarandus caribou)*	Yes	EN	X	X		MicroSattelite	Thompson et al., 2019	Smaller populations (driven by climate change) show genetic erosion
Southern brown tree frog *(Litoria ewingii)*	Yes	LC	X	X		MicroSattelite	Potvin et al., 2017	Erosion after vast population decline due to fire
Victorian frog *(Litoria paraewingi)*	Yes	LC	X	X		MicroSattelite	Potvin et al., 2017	Erosion after vast population decline due to fire
Lapland ringlet (*Erebia embla)*	Yes (temporal)	NT	X			hyRAD	Gauthier et al., 2020	
Violet copper (*Lycaena helle)*	Yes (temporal)	EN	X			hyRAD	Gauthier et al., 2020	
Iberian lynx *(Lynx pardinus)*	Yes	EN	X		X	WGS	Abascal et al., 2016; Marmesat et al.,2017	
Pygmy hog *(Porcula salvania)*	Yes	CE	X		X	WGS	Liu et al., 2020	
Common hamster *(Cricetus cricetus)*	No (temporal)	CE				MicroSattelite	Reiners et al., 2014	
Southern white rhinoceros *(Ceratotherium simum simum)*	Yes (temporal)	NT	X	X		WGS	Sánchez Barreiro et al., 2020	
Northern white rhinoceros *(Ceratotherium simum cottoni)*	Yes (temporal)	EW	X	X		WGS	Sánchez Barreiro et al., 2020	

Huia (*Heteralocha acutirostris*)	No	EW				WGS	Dussex et al., 2019	Fast population decline after predator introduction
Kokako *(Callaeas cinereus)*	No	EW				WGS	Dussex et al., 2019	
North sea cod *(Gadus morhua)*	Yes (temporal)	VU				MicroSattelite	Hutchinson et al., 2003	
Przewalski's horse *(Equus ferus przewalskii)*	Yes (temporal)	EN	X	X		WGS	Der Sarkissian et al., 2015	
Squirrel glider *(Petaurus norfolcensis)*	Yes	LC	X			MicroSattelite	Taylor et al., 2011	Population decline due to habitat fragmentation
Common ringtail possum *(Pseudocheirus peregrinus)*	Yes	LC	X		X	MicroSattelite	Lancaster et al., 2011	Population decline due to habitat fragmentation
Snow leopard *(Panthera uncia)*	Yes	VU	X	X		WGS	Cho et al., 2013	
Red-backed salamanders *(Plethodon cinereus)*	Yes	LC	X		X	MicroSattelite	Noël & Lapointe, 2010	Population decline due to urbanization
Far eastern/Amur Leopard *(Panthera pardus orientalis)*	Yes	CE	X	X		MicroSattelite	Uphyrkina et al., 2002	
African cheetah (*Acinonyx jubatus*)	Yes	VU	X	X		WGS	Dobrynin et al., 2015	
Tasmanian devil (*Sarcophilus harrisii*)	Yes	EN	X		X	Microsattelite and MHC genotyping	Morris et al., 2015; Siddle et al., 2007	
Rhinella ornata	Yes	LC	X			BigDye sequencing	Dixo et al., 2009	Population decline due to habitat fragmentation
Cheilotoma musciformis	Yes	EN	X			BigDye sequencing	Kubisz & Mazur, 2013	
Common wall lizard (*Podarcis muralis*)	Yes	LC	X			MicroSattelite	Michaelides et al., 2015	Population decline due to habitat fragmentation
Corsican red deer (*Cervus elaphus corsicanus*)	Yes	EN	X			MicroSattelite	Hmwe et al., 2006	
Kashmir red deer (*Cervus elaphus hanglu*)	Yes	CE	X	X		MicroSattelite	Kumar et al., 2015	
Malawi population of Giraffe (*Giraffa giraffa giraffa*)	Yes	VU	X	X		MicroSattelite	Winter et al., 2019	Reintroduced in Malawi, genetic profiling showed the population to consist of South African Giraffes
Large blue butterfly *(Maculinea arion)*	No (temporal)	NT				MicroSattelite	Ugelvig et al., 2011	Small population due to habitat fragmentation
Spanish imperial eagle (*Aquila adalberti*)	No (temporal)	VU				MicroSattelite	Martinez-Cruz et al., 2007	Population decline due to habitat fragmentation (only mtDNA differed)
Hispaniolan solenodon *(Solenodon paradoxus paradoxus)*	Yes	LC	X			WGS	Grigorev et al., 2018	Considered endangered u/i 2020
Hispaniolan solenodon (*Solenodon paradoxus woodi*)	Yes	LC	X			WGS	Grigorev et al., 2018	Considered endangered u/i 2020
Scalloped hammerhead shark (*Sphyrna lewini*)	Yes	CE	X			MicroSattelite	Quintanilla et al., 2015	
Baji/Yangtze river dolphin *(Lipotes vexillifer)*	Yes	CE	X	X		WGS	Zhou et al., 2013	
Naked mole rat *(Heterocephalus glaber)*	Yes	LC	X	X		WGS	Kim et al., 2011	
Cranberry fritillary butterfly *(Boloria aquilonaris)*	Yes	LC	X	X		MicroSattelite/ RAPD/ Allozymes	Turlure et al., 2014	Small population due to habitat fragmentation
Mountain yellow-legged frog *(Rana muscosa)*	Yes	EN	X			MicroSattelite	Schoville et al., 2011	
Montezuma quail *(Cyrtonyx montezumae)*	Yes	VU	X			WGS	Mathur et al., 2019	Considered vulnerable in this specific country
